# Mothers’ and fathers’ lived experiences of postpartum depression and parental stress after childbirth: a qualitative study

**DOI:** 10.1080/17482631.2020.1722564

**Published:** 2020-01-28

**Authors:** Maude Johansson, Ylva Benderix, Idor Svensson

**Affiliations:** Department of Psychology, Linnaeus University, Växjö, Sweden

**Keywords:** Postpartum depression, parental stress, child health care, parental support, qualitative research

## Abstract

**Purpose**: The study aims are to explore the lived experiences of mothers and fathers of postpartum depression and parental stress after childbirth.

**Methods**: Qualitative interviews conducted, and analysed from an interpretative phenomenological analysis (IPA) perspective.

**Results**: Both mothers and fathers described experiences of inadequacy, although fathers described external requirements, and mothers described internal requirements as the most stressful. Experiences of problems during pregnancy or a traumatic delivery contributed to postpartum depression and anxiety in mothers and affected fathers’ well-being. Thus, identifying postpartum depression with the Edinburgh Postnatal Depression Scale, mothers described varying experiences of child health care support. Postpartum depression seemed to affect the spouses’ relationships, and both mothers and fathers experienced loneliness and spouse relationship problems. Experiences of emotional problems and troubled upbringing in the parents’ family of origin may contribute to vulnerability from previous trauma and to long-term depressive symptoms for mothers.

**Conclusions**: The findings of this study demonstrate the significant impact of postpartum depression and parental stress has in parents’ everyday lives and on the spouse relationship. These results support a change from an individual parental focus to couples’ transition to parenthood in child health care.

## Introduction

Already in the late ’90s, Kirby Deater‐Deckard (1998) established that parenting stress linked to adult functioning, the quality of the parent-child relationships, and child functioning. Furthermore, research has established a link between postpartum depression and parental stress, concluding that postpartum depression is the most reliable predictor for parental stress (Leigh & Milgrom, 2008).

Many research studies have found that postpartum depression in mothers is common after delivery, with a prevalence rate ranging from 10% to 15%, depending on the criteria used for diagnosis (Brummelte & Galea, 2016; Shorey et al., 2018). Meanwhile, prenatal and postpartum depression was evident in about 10% of men and was relatively higher in the 3- to 6-month postpartum period (Paulson & Bazemore, 2010).

Prevalence studies on depression following delivery have primarily focused on mothers, and generally on the period of the first year after birth. However, a Swedish study found the prevalence of depressive symptoms, to be 11.3% for the mothers, and 4.9% for the fathers 25 months after childbirth (Johansson, Svensson, Stenström, & Massoudi, 2017).

Research in postpartum depression and parental stress in fathers showed that fathers are as well as the mothers affected by the same types of mood alteration during the transition to parenthood and that their mental health has a significant impact on the child’s development and the health of the family (Goodman, 2004; Ramchandani, Stein, Evans, & O’Connor, 2005). Especially postpartum depression in fathers has been noted to exacerbate the effects of maternal depression on a child’s behavioural problems (Mezulis, Hyde, & Clark, 2004; Paulson, Dauber, & Leiferman, 2006).

Parental stress defined as psychological distress arising from the demands of bringing up children. While most parents experience some degree of parental stress, some experience in terms of a feeling of significant aversion and negativity towards themselves and their children (Webster-Stratton & Hammond, 1988) marital quality (Kerstis, Engström, Sundquist, Widarsson, & Rosenblad, 2012) the quality of parenting behaviour, and child adjustment (Elgar, Mills, McGrath, Waschbusch, & Brownridge, 2007).

A recent review suggests that the timing of maternal distress (which includes anxiety, stress, or depression) has different consequences for the child’s development (Kingston, Tough, & Whitfield, 2012) and indicates that maternal distress becomes even more critical for the child’s development and social behaviour after the first year (Gjerde et al., 2017; Weissman, 2016). Research in postpartum depression has stated the importance of good mental health in both parents as a prerequisite for the development of good child behaviour and parent-child relationship (Schumacher, Zubaran, & White, 2008; Sroufe, Coffino, & Carlson, 2010).

Most of the studies conducted internationally and in Sweden have been quantitative and have identified the prevalence of postpartum depression symptoms and risk factors for mothers or fathers postpartum. Risk factors for postpartum depression are problems in the partner relationship, previous depression, stressful life events, and low partner support (Kerstis, 2015; Massoudi, Hwang, & Wickberg, 2016; Paulson & Bazemore, 2010; Psouni, Agebjörn, & Linder, 2017). The studies support the clinical standpoint that advocates the need for identifying and treating postpartum depression in parents of infant children (Brummelte & Galea, 2016).

However, there is a gap in the research, and a lack of scientific studies focuses on both mothers’ and fathers’ experiences of postpartum depression and parental stress.

The study aims are to explore the lived experiences of mothers and fathers of postpartum depression and parental stress after childbirth

## Methods

The study is rooted in an Interpretative Phenomenological Analysis (IPA). The choice of IPA is especially suitable when the researcher seeks a rich and detailed description from a homogenous group of informants on their lived experience in a phenomenon (Smith, Flowers, & Larkin, 2009). IPA focuses on how individuals describe the phenomenon, and the author develops a description of the experience about the phenomenon. In IPA, a double hermeneutic is involved as a two-stage interpretation process. The participant trying to make sense of their world, and the researcher is trying to make sense of the participants’ sense of their world. IPA connected to hermeneutics and theories of interpretation (Smith & Osborn, 2008). It addresses different aspects of this lived experience ranging from individuals’ wishes, desires, feelings, motivations, and belief systems through to how these translated into behaviour and action. In phenomenology, it is essential for the researcher, as the primary research instrument, to be aware of their preconceptions and beliefs as these could influence the research process. It is not humanly possible for qualitative researchers to be objective. To minimize research bias in phenomenological research uses’ bracketing’, which means that the researcher must have a reflective approach through the whole research project and identify areas of potential bias to minimize their influence by bracketing them. The researcher is supposed to use fresh eyes and to set aside preconceived attitudes, beliefs, or opinions.

### Settings and participants

This study is part of a larger study.

The data collection method is based on the fact that parents who visited the Child Health Centre, and who have experienced depressive symptoms and parental stress had to report their interest to participate in the study. It was voluntary to participate and no pressure was made to get more parents to participate. After one year, fifteen parents had accepted to participate and the researchers determined that saturation had occurred as the parents had given rich and deep description of their experience of depressive symptoms and parental stress.

In the study it was ten mothers and five fathers, given their consent to participate.

#### Data collection

The parents were asked to participate in the study when they visited the Child Health Care Nurse for the 2.5 years visit. Some parents lived in the city and some of them were living in the country side. The mean age for the mothers was 30 years (25–40) and for the fathers 35 years (30–43). The numbers of children for the mothers were 1–4 and for the fathers 1–3. Seven of the mothers had a University education, and three had College. For the fathers, four had a University education, and one had College. All fathers worked full time, three had employment, and two were self-employed. Five of the mothers worked full time, three mothers worked 75%, one mother was on parental leave, and one mother was on sick leave. Two of the participants were a couple.

Before the interviews, a schedule with non-directive, open-ended questions were prepared. The interview schedule was not strictly followed, instead it was adapted to the parents’ narratives and included in the process of reflecting and probing of what was important, for example, by responding: ‘you said that … tell more about that …, what were the feelings. The interviews lasted between 45 and 90 minutes. (See Table 1)

**Table 1.** Interview guide

**Background factors**	Age Number of Children? Relation to the Childs, other Parent? Parental Leave? Work?
**Relation to parents and friends**	Tell about childhood/ and young adult? Describe mental health during childhood and young adult? Describe the relation to the parents after becoming a parent? Describe relations to friends during childhood and as an adult? Tell me about your support everyday
**Pregnancy/ Childbirth**	Tell me about your pregnancy Tell me about the delivery Tell me about health after becoming parents?
**Treatment/support?**	Did you have treatment or support for your problems? Was the treatment or support helpful? Tell me about your experiences of CHC? Did you have any discussion about your health with the Nurse in CHC Did you do EPDS – screening for Postpartum depression?
**Relation to the other parent?**	Tell me about your relationship to Spouse? Describe the relationship after delivery?
**Work**	Tell me about work situation?

#### Ethics approval and consent to participate

The study received ethical approval from the Regional Ethics Review Board in Linköping, Sweden (Register number 2016/193-31), and performed by the Helsinki Declaration. All research procedures conducted following the requirements of the Regional Ethics Review Board in Linköping, Sweden, including the informed consent of participants. The parents who provided their contacts, was contacted by phone or email for an interview.

### Analysis

The interviews were recorded digitally and transcribed verbatim. The interview transcripts were analysed using IPA by following five steps, as described by Smith (2015). The first stage involved becoming familiar with the transcript and noting any essential aspects, observations, and preliminary interpretations. Then emerging themes were noted and transformed into more specific themes, which were clustered by connecting them, followed by capturing the main categories of meaning conveyed by the participants.

A descriptive label was given to each cluster to express the conceptual nature of the themes involved (Smith, 2015). At the end of the process, a summary of the higher-order themes was conducted. The first and the second author read the transcripts and developed the thematic framework independent of each other. The two authors also decided together which themes best described the parents’ lived experiences. Finally, the third author read the themes and analyses to ensure that the meaning of the participants’ narratives was significant.

Since many themes emerged from individual transcripts, those that appeared in at least half of them were categorized as current themes to promote an ideographic perspective while simultaneously counterbalancing a more generic perspective. The IPA research methodology adapted to an interpretive approach with attention paid to the complexity and context of the participants’ experiences in order to obtain a rich understanding of their perspective (Smith et al., 2009).

The participants provided information about their experiences of postpartum depression and parental stress in in-depth interviews in which the subjects asked retrospective questions concerning their experiences of postpartum depression and parental stress after childbirth. The main themes or the essence in the study, found in the interview were experiences of inadequacy resulting from external or internal requirements, problems in pregnancy or a traumatic delivery, varying experiences of child health care support, spouse relationship problems, and vulnerability from previous trauma (See Table 2).

**Table 2.** Participants experiences during first years after childbirth

		Complicated pregnancy	Mental wellbeing	Spouse relt.	Support
Parents	Co-habit	Traumatic delivery	Postpartum	Problems	CHC
Mother 1	Yes	Loss of own father during pregnancy	High stress	Yes	No
Mother 2	Yes	Early labour/unprepared	Depressive	No	No
		Immigrant/the husband			
Mother 3	Separated	Partner left during pregnancy	Depressive	Yes	Yes
Mother 4	Yes	Early labour/ Neonatal care	Depressive	No	No
Mother 5	Yes	Acute Caesarean	Depressive/anxiety	No	No
Mother 6	Yes		Depressive	Yes	Yes
Mother 7	Separated	Complicated labour/Caesarean v 36	Depressive	Yes	No
		Neonatal Care			
Mother 8	Separated		Depressive	Yes	No
Mother 9	Yes	Complicated labour/IVF	Depressive/anxiety	No	Yes
Mother 10	Separated	Complicated labour/	Depressive	Yes	Yes
		Caesarean for all children			
Father 1	Separated	Emergency Caesarean		Yes	No
Father 2	Yes	Complication after	High Stress	No	No
		Delivery			
Father 3	Yes	Complicated Pregnancy	High Stress	Yes	No
Father 4	Yes	Three miscarriages	High Stress	No	Yes
Father 5	Separated		High Stress	Yes	No

### Findings

#### Inadequacy resulting from external or internal requirements

In the interviews, the mothers and fathers provided different narratives of their experiences of parental stress, although both described an underlying feeling of inadequacy. Fathers talked about external requirements such as work, economy, compensation for being off work to care for a sick child i.e., (VAB) or childcare restrictions, caused stress in their everyday lives. Most fathers expressed stress in their parental role concerning shared parenthood and the demands of working life, as well as when the child was sick or if meetings delayed. These were all stressful matters, but coping with childcare restrictions was especially so.

We work a lot, both of us, and then VAB is very stressful
The childcare organization does not work, childcare does (4)

The fathers’ talked of importance participating in their children’s lives, and all were engaged in their daily care, such as taking the child to and from day-care and applying for VAB so that they could stay home if the child were sick. All fathers had taken parental leave, one father for 14 months, another for more than six months, and one father had even taken it along with full shared custody after separating from the child’s mother.

I will be different from my father, and I want to be part of
my daughter’s life in every respect from Saint Lucy’s Day
to doctor visits (1).

The mothers expressed their parental stress concerning internal requirements concerning their children, their families, and the domestic situation. They described their well-being as going into a downward spiral, feeling overwhelmed by the responsibilities of caring for their families.

Sometimes I have been stressed to pieces,
and on sick leave, I have been overwhelmed by
my demands, and it felt like I had sole responsibility
for the child. (10)

Many of the mothers perceived parenting as their responsibility, and displayed a distressing range of emotions, including sadness, anger, guilt, feeling overwhelmed, anxiety, loneliness, and a sense of shouldering the overall responsibility for the family. Furthermore, for some mothers, this was not merely a feeling. It became apparent from their narratives that they quite literally carried the responsibility for taking care of the children and the household. The spouse was absent, often working or renovating the house.

I drowned in all the requirements, and my husband
worked much. I felt that I had the responsibility for the child (7).

Some of the mothers expressed shame and helplessness, both in their relationship and in their situation more generally, and had noticed a change in their spouse since he had become a father; he would be increasingly absent, and this affected them, as mothers, because they felt abandoned and subsequently blamed themselves.

I was ashamed and felt that I had chosen.
the wrong partner, a partner who does not
do his bit (8).

#### Problems in pregnancy or a traumatic delivery

When asking the parents about their well-being after childbirth, the fathers talked about feelings of stress in everyday life and finding a balance between their employment responsibilities and coping with the new parental role. Fathers’ well-being, were affected, but none of them stated that they had felt depressed, even though the fathers revealed that there had been complications during their wives’ pregnancies and deliveries and that these had affected them.

It has been tough for us with three miscarriages.
When looking back, we have not been feeling
very well (4).

The mothers, on the other hand, stated very clearly during interviews that they had been depressed—all except one who described parental stress. Many of the mothers revealed that they had become depressed almost immediately after birth.

I started feeling sad in the maternity ward (2).

One mother had had several miscarriages before having a child. She described feeling a great deal of fear over losing the baby during every pregnancy. After childbirth, she became depressed and experienced a lot of anxiety and guilt for not feeling well.

I had so much guilt for giving birth
to this child that I have fought so much
for, and then I was depressed (9).

Several mothers in the study had experienced complications during pregnancy and traumatic delivery. For many of them, it seems that the traumatic childbirth had not processed and that they had a great need to talk about it and how it had affected their well-being. Moreover, mothers reported experienced difficulty connecting emotionally to the child and that these feelings were hard talking about with the spouse, CHC, or relatives, so their coping strategy had been to struggle with their feelings alone. The mothers described how they had tried to compensate for the absence of positive feelings by excessively focusing on the child.

The pregnancy was tough. I may have felt
a bit low, even before delivery.
I did not think the baby was pretty, and
I had no feelings of love (10).

Some of the mothers had experienced a traumatic delivery with acute caesarean section and multiple miscarriages had developed anxiety symptoms. This anxiety could take expression in the form of panic attacks when alone with the baby or of social phobia. The mothers with this condition did not tell their spouse, relatives nor the CHC nurse but kept it to themselves.

I have had many panic attacks
after the delivery (5).

Some of the mothers experienced loneliness because the fathers had to leave the hospital, due to lack of space or because of a sick child, if it was matched with an experience of a complicated pregnancy or delivery, it affected the mother’s well-being, causing anxiety and a feeling of not being able to cope with the situation on her own.

After the delivery, my husband was not
allowed to stay in the maternity ward I
was dead tired and was supposed take
care of this new thing that does nothing but cry.
It felt strange to ring a bell and say that you do not quite
know what to do with the child (9).

#### Varying experiences of child health care support

Both the mothers and fathers described varying experiences of child healthcare support. Three fathers expressed a lack of support.

Help from the CHC is non-existent,
in my opinion. You get a quarter of an hour.
That is, it. Nobody asks how things are or how
you feel; moreover, nobody has time to listen (2).

One father, who had had the most contact with the CHC for his child, commented on the nurse concerning asking about how he felt,

No, the nurse did not ask me, we did not talk
about my health (3).

While another father concluded that the support was

Very good during a difficult time with
three miscarriages (4).

All of the mothers in the study had performed the Edinburgh Postnatal Depression Scale (EPDS, screening for postpartum depression), and several of the mothers had negative experiences about the subsequent support they received. The mothers described scoring highly in the questionnaire but not being offered help afterwards.

I was told to fill in a paper at CHC.
The nurse looked at the paper and said
that it showed that I was depressed,
no more response (8).

The mothers had opinions about how the EPDS questionnaire used in the subsequent talk about their well-being. The mothers had had higher expectations about the talk after doing the questionnaire, primarily if they had scored highly, indicating that they were depressed.

EPDS, yes, I have done that twice,
but I felt that the nurse, did not have
so much knowledge (2).

The mothers with negative experience of EPDS, despite high scores described that they didn’t get any help for their depressive symptoms. On the other hand, it was mothers in the study describing that they had received excellent support from the CHC. These mothers emphasized the importance of the CHC, the support provided by the nurse, and how well the nurse had understood that they were depressed. These mothers described receiving excellent professional support.

I had a beautiful nurse, and I did not need
to say that I did not feel well.
The nurse said, “How are you?” moreover,
when I said, “Fine,” she said, “Really?” (6)

For most of the mothers, it was difficult to admit and talk about

postpartum depression. One mother had not talked with a CHC nurse or her husband about her anxiety. It was after she conducted the questionnaire for the study, two-and-a-half years after the delivery that she decided that she needed and has to ask for help for her panic attacks.

It may be a problem for CHC if the mothers are avoiding talking about their depressive symptoms, alternatively, other difficulties concerning parenthood and family life when seeing CHC nurses.

It is a big step, but I was honest the last time,
and the nurse said, “You are not feeling well?” (6).

The mothers that had experienced depression also described feelings of shame and guilt. They had moral beliefs regarding the definition of a “good mother” and that she should always think of her child first. Although they had placed all their focus on the child’s well-being, and there was nothing in their descriptions that suggested neglect, they nevertheless described feelings of inadequacy. One mother, for example, related her feelings and explained why she had not discussed them with the CHC nurse:

To give birth to a child and not be able to
take care of it, what kind of person am I then? (1).

For some mothers, however, the depressive symptoms transitioned from sadness to irritation, and even aggression, and this change caused them to realize the seriousness of the situation and induced to seek help.

After a while, my sadness became aggression.
I became angry when I was by myself with the children,
and feared I would spank them (8).

#### Spouse relationship problems

Most of the mothers described the relationship with their partners as having become strained after being made parents. They had felt abandoned by their partner and expressed feelings of loneliness and disappointment because they perceived that the husband had changed after childbirth, and this had affected the relations.

I have felt bitter towards my husband because I do not
think that he supported or pulled his weight correctly, and then
it feels like even if you have got time for each other,
I felt no desire (6).

The mothers had forced taking full responsibility for the children and had felt ashamed or angry at not having a partner who was committed to their child or to supporting his wife, which was one of the reasons some of the couples subsequently separated.

The fathers’ narratives about their well-being after birth also seemed to affect the spousal relationship; a lack of intimacy and sexual desire put a strain on the marriage and also resulted in separation.

No intimacy. We did not have any
sexual relations (1).

Of the participants in the study, two couples interviewed. In one of the couples, the husband talked about how difficult the experience of his wife’s depression was and how it affected his well-being.

It is tough in many ways. It is also doubly sometimes
I feel very sorry for her, but sometimes I get provoked
and irritated. I know this is wrong, but I feel so anyway (3).

#### Vulnerability from previous trauma

Several mothers experienced spousal relationship problems, with being emotionally and physically abandoned after becoming a parent. Many of the mothers also described similar experiences with their family of origin, including restricted emotional and practical support and, for some mothers, outright neglect.

I did not have a wonderful childhood. I have
always thought that I should never become like
my mother. (10)

Some mothers revealed they abandoned emotionally and physically in their childhoods, such as being left alone to take care of themselves and their siblings. Some mothers had additionally experienced a lack of emotional security or a harsh upbringing.

Mother wanted to live her own life. She was a young
mother, so she liked partying and left us on our own.
She drank a lot, so I felt abandoned (9).

## Discussion

The study aims were to explore the lived experiences of mothers and fathers of postpartum depression and parental stress after childbirth.

The first theme was mothers’ and fathers’ feelings of inadequacy and that mothers and fathers provided different narratives of their experiences. The fathers expressed their inadequacy as stress while the mothers expressed their inadequacy as depressive symptoms. In this regard, the fathers expressed external requirements in connection with their working lives, while the mothers expressed internal requirements related to parenthood. The mothers and fathers described inadequacy and a sense of a lack of fulfilment in both work and parenthood. The fathers in the study were engaged in their children’s lives but described their particular feelings of inadequacy as being due to a demanding career, making it challenging to combine work, childcare, and a demanding schedule. The mothers, on the other hand, experienced parental stress in the form of internal requirements concerning the child, the family, and the domestic situation as well as an overwhelming feeling of responsibility, all of which led to feelings of inadequacy and depressive symptoms. It was evident from the mothers’ narratives that they had high expectations of themselves to be “a good mother”—one who could cope with the family situation while simultaneously being happy and content. The mothers expressed shame and guilt at not feeling happy and content with their situation. The fathers in our study did not identify themselves as being depressed, although there were signs in their stories that indicated that they had experienced depressive symptoms, the fathers seemed to express their stress and negative feelings in irritation. Some of the fathers in the study seemed to regulate their stress and irritation through work and to reduce family involvement. In a meta-study about men’s help-seeking behaviour, the researchers observed that a growing body of gender-specific studies had highlighted a trend in men delaying seeking help when they became ill (Galdas, Cheater, & Marshall, 2005) and a review in men’s health found that men are less likely to employ healthy, vigilant coping strategies and to acknowledge that they need help (Courtenay, 2003). The mothers identified themselves as depressed, none of the fathers, despite this, mother didn’t talk with their partner and some of the mothers had also difficult to reveal for the CHC nurse about their feelings. It indicates that postpartum depression is a “silent” condition difficult to talk about and especially at a time when parents are expected to be happy. It can also be hard for parents to understand that their feelings of irritation and unhappiness may depend on postpartum depression requiring treatment. It is therefore important to inform new parents about symptoms included in the conditions. In a review about prevalence of perinatal depression, (during pregnancy and one year after delivery) have found that about 12% develop postpartum depression after child birth (Woody, Ferrari, Siskind, Whiteford, & Harris, 2017). It is not unusual that if one partner is depressed, the other is also affected (Goodman, 2004) and if both parents are affected it is even more important to offer treatment.

The second prominent theme concerned problems during pregnancy or a traumatic delivery, many of the parents in this study experienced complications during pregnancy or had a traumatic delivery, and this seemed to have led to postpartum depression, and anxiety, sometimes accompanied by panic attacks for the mothers and negative well-being, for the fathers. Experiencing such problems during pregnancy or delivery may represent risk factors for subsequent postpartum depression or anxiety. These relations have also identified in another study, which found that PTSD was associated with the delivery, with depressive illness, and with difficulties in the mother-infant attachment (Ballard, Stanley, & Brockington, 1995; Söderquist, Wijma, Thorbert, & Wijma, 2009).

This further highlights the third theme: varying experiences of child healthcare support. All mothers in the study were screened with the EPDS to identified postpartum depression. Many of the mothers identified with high scores had excellent support but some of the mothers did not get any support or treatment at all, especially when the CHC nurse had not taken responsibility themselves for supporting and treating the mothers with postpartum depression. Maybe this was due to a lack of time on behalf of the CHC staff having too many tasks to fulfil (Wells, Massoudi, & Bergström, 2017) or whether the nurses had no treatment to offer. It highlights the importance of the availability of CHC services and the need for professionals to have enough time to dedicate to both the mother and father and the option to provide education, counselling and support to parents experiencing stress and postpartum depression.

The American Psychiatric Association (APA) has emphasize the importance of identifying, educating, and following up with mothers from the stages of pregnancy up to six months after delivery with repeated screenings (Actions, 2018). In this study, it seems that if a mother’s postpartum depression and parental stress remain untreated, she is at risk of developing enduring problems that affect not only herself but also her relationships with her child and spouse; this is also in line with other studies (Lung, Shu, Chiang, & Lin, 2009; Milgrom & McCloud, 1996).

The fourth theme was loneliness and spousal relationship difficulties. Both mothers and fathers experienced that postpartum depression affected the relationship with the spouse and that both mothers and fathers expressed feelings of loneliness and spousal relationship problems. The mothers reported loneliness in parenthood and had feelings of taking on sole responsibility for the household and the child. It affected spouses’ relationship with the mothers and some fathers, noting difficulties with intimacy and emotional closeness with their spouse, which for some, even led to a separation. None of the fathers talked about receiving support for these relationship issues or themselves. On the other hand, mothers wanted help for themselves and as a couple. It seems as the fathers in this study, as well as the mothers see their problems, but not as something to seek help for maybe as something that they hope to solve for themselves. Galdas et al. (2005) found in there review that men were less likely than women to seek help for depression, stressful life events, or physical problems. If fathers can obtain information about postpartum depression during pregnancy and in the first years of parenthood, maybe it would be easier for them to seek treatment, this may help to reduce suffering for fathers with parental stress or postpartum depression. Results from recent studies have concluded that fathers’ mental health has a significant impact on the father-child relationship and the child’s development (Ramchandani et al., 2005; Schumacher et al., 2008) as well as on the mothers’ well-being and the spousal relationship (Hanington, Heron, Stein, & Ramchandani, 2012; Kerstis et al., 2012). It indicates the necessity of identifying the father’s well-being as much as that of the mother. The mothers expressed their feelings of depression frankly and clearly, including feelings of inadequacy, shame, and guilt due to a variety of reasons, especially for being a “bad mother.” Findings like these have been reported in other studies where mothers expressed their isolation and loneliness and withheld these feelings from family members (Beck, 2002). The mothers’ openness about their depression in this study may have been because they had not experienced postpartum depression for some time and could, therefore, reflect more openly on their previous situation.

The final theme was vulnerability due to previous trauma led to long-term problems. Many of the mothers described their upbringings as involving neglect, insecurity, and emotional abandonment, which may have created a vulnerability for depression upon becoming a parent. These results were consistent with the psychological knowledge that a troubled upbringing may constitute a risk factor for later depressive symptoms and parental stress (Ethier, Lacharite, & Couture, 1995; Paley et al., 2005; Røseth, Bongaardt, & Binder, 2011; Weissman, 2016).

For one mother, her distress after becoming a parent lasted for more than a decade and for another mother, and it had taken her nearly years to understand that she needed treatment and support for her depressive symptoms and traumatic childhood. For two mothers with depressive symptoms in adolescence, these symptoms recurred when they became parents. All these mothers displayed enduring problems affecting not only themselves but also their spouses and their relationship with their child. The experience of a troubled upbringing seems to affect mothers’ psychological well-being and puts them at risk of developing postpartum depression. Weissman, (2016) identified that the offspring of depressed parents represent a high-risk group for psychiatric and medical problems that begin early on and continue through adulthood, eventually leading to spousal relationship problems (Cummings, Keller, & Davies, 2005; O’hara & Swain, 1996).

Parenthood today requires the participation of both parents from childbirth onwards. Furthermore, there is a considerable body of evidence that points to the fundamental importance of the adult couple’s relationship and how it has a significant impact on their own and their children’s mental and physical well-being.

### Strengths and limitations

Qualitative research has its built-in limitations, for example, causal conclusions. It was not possible to draw any specific causal conclusions between the parent’s experiences and the development of postpartum depression and parental stress. However, the narratives offered a nuanced picture of their individual experiences, reminding them about other studies of parent’s feelings of depressive symptoms and parental stress (Johansson, Svensson, Stenström, & Massoudi, 2017; Kerstis et al., 2012; Widarsson et al., 2013).

The sample is not demographically represented, as most of the parents who accepted to participate in this study were highly educated and had employment. This limitation has also given the study the rich data as the parents had an excellent ability to describe their experiences.

The study did not include the same number of fathers and mothers as the study contents the number of parents that accepted to participate during the year of data collection in the study. This is a limitation of the study, that the numbers of fathers are rather few and might jeopardize the saturation. Thus, all the fathers in the study described the phenomenon of parental stress and inadequacy in the same way, and that may be the essence of the lived experience of parental stress for Swedish talking, highly educated fathers with a demanding work situation after childbirth.

In our study, parents who had experienced depressive feelings or/and parental stress were invited to participate in an interview about their experiences. As the phenome depression is a sensitive subject to talk about it is difficult to get especially fathers to be part of an interview about these conditions. It was not possible to get more fathers to participate during the study. Despite it was fewer fathers then mothers the fathers described their experiences of the phenome similar and therefore we think that it was possible to compile to credible themes also for the fathers. Another implication is that Sweden is still is rather homogeneous culturally and economically. This means that the fathers in the study are a homogeneous group and the meta-themes that have emerged can be said to fulfil saturation for this particular group (well-educated and with demanding work). However, the meta-themes cannot be generalized to immigrant groups, nor can we generalize to fathers who are uneducated and without work.

There are many challenges to determining the appropriate size needed for purposeful samples used often in qualitative research especially when the subject is mental illness. In a qualitative sample, individuals, objects, events, or settings are often purposively selected according to relevant predetermined criteria (Sandelowski, 1995)

Another limitation was that no immigrant mothers accepted to be included in the study and the results may not include the same experiences for immigrant mothers.

This may need an own study about immigrant mothers and fathers’

## Implications

To create a more gender-equal society, Sweden has enacted a family policy that supports mothers and fathers sharing child-rearing duties. Swedish fathers are expected to be involved in the caring early in their child’s life. Much research concerning difficulties on the transition to parenthood as a risk factor for postpartum depression and parental stress in fathers’, CHC support is organized in a way primarily suits mothers’ (Wells et al., 2017).

CHC is an essential support for parents. The nurses have many tasks to fulfil, including health care, screening, educating parents, and giving support for parental psycho-social issues. This diversity and high workload may make it challenging to identify and give support to mothers and fathers with postpartum depression, as these conditions require a good relation, time to give regular support, and professional competence to work with both parents (Wells et al., 2017).

EPDS identifies parents with postpartum depression but, as one mother said: “It is more than putting a cross [in a box].” A great many parents may not meet the cut-off point, but may still need support to prevent depression later during the child’s preschool year and parental stress are seldom an exclusively individual problem as they affect not only the depressive parent but also the spouse and the child (Cummings et al., 2005; O’hara & Swain, 1996).

Gender equality requires that fathers and mothers have the same opportunity to develop in their parenthood but also necessitates a systematic approach to supporting the parents, which involves focusing on the whole family. Parenthood requires the participation of parents from delivery and onwards, and a CHC organized answering to these demands and needs for parent support, which has to take into consideration in the treatment. It is, therefore, essential that the CHC sees both parents together since being a parent requires participation from both parents right from the delivery.

## Conclusion

The findings of the current study demonstrated the significant impact parental stress and depressive symptoms have on the parents’ everyday life and the importance of health professionals’ knowledge to identify and support parents with these conditions. Not only during the first year after birth but throughout infancy and also include the other parent. In our knowledge, no past research has focused on the specific implications of both mothers’ and father’s experiences of postpartum depression, parental stress after the first year of childbirth.

Acknowledging these experiences and their implications will improve interventions and support by health professionals as they assist families to adjust to parenthood, not only on an individual level but also as a couple. Reduction of the duration of postpartum hospital stay in Sweden highlights the need for better support and continuity of care for expectant and new mothers (Barimani & Hylander, 2012).

Future research should examine the role of CHC nurses and their experiences of supporting parents with postpartum depression and parental stress.
